# Immunomodulatory Effects of a Tick Salivary Serpin on Psoriasis-like Inflammation

**DOI:** 10.3390/life16030427

**Published:** 2026-03-06

**Authors:** Mohamed Amine Jmel, Huimei Wu, Constance C. F. M. J. Baaten, Xueqing Xu, Kutty Selva Nandakumar, Michail Kotsyfakis

**Affiliations:** 1 Institute of Parasitology, Biology Centre, Czech Academy of Sciences, 37005 České Budějovice, Czech Republic; amine.jmel@gmail.com; 2 Université de Lorraine, CNRS, IMoPA, F-54000 Nancy, France; 3 School of Pharmaceutical Sciences, Southern Medical University, Guangzhou 510515, China; 4 Department of Biochemistry, Cardiovascular Research Institute Maastricht, Maastricht University, 6200 MD Maastricht, The Netherlands; 5 Institute for Molecular Cardiovascular Research, University Hospital Aachen, RWTH Aachen University, 52074 Aachen, Germany; 6 Institute of Molecular Biology and Biotechnology, Foundation for Research and Technology-Hellas, 70013 Heracklion, Crete, Greece

**Keywords:** *Ixodes ricinus*, immunomodulation, serpins, skin inflammation, psoriasis

## Abstract

Psoriasis is a chronic inflammatory disease with a complex pathogenesis, and it is mainly driven by a dysregulation in immune responses. Therapeutic strategies constantly require novel compounds targeting immune modulation to substitute the current traditional drugs characterized by side effects and limited efficacy. In this study, we used a mannan-induced psoriasis-like inflammation mouse model to investigate the immunomodulatory potential of Iripin-3, a salivary serpin from the *Ixodes ricinus* ticks. Mice treated with Iripin-3 showed improvements in the severity of psoriasis-like lesions, as shown by the psoriasis area severity index (PASI) scores, epidermal thickness, and baker’s scores. Iripin-3 modulated the immune cascade by inhibiting dendritic cells and γδ T cells expression in secondary immune organs while increasing macrophages and neutrophils in skin. On the other hand, Iripin-3 exhibited significant reductions in the expression of inflammatory cytokines such as TNF-α, IL-22, IL-23, and IL-17 family cytokines, indicating broad immunomodulatory effects. Our findings suggest that Iripin-3 offers a unique and targeted mechanism of action through modulation of the IL-23/γδ T/IL-17 axis involved in mannan-induced psoriasis-like inflammation and thus could be a promising therapeutic candidate for treating psoriasis. Further studies are required to explore its translational potential in wider clinical settings.

## 1. Introduction

Psoriasis is a chronic inflammatory skin disease that affects up to 2–3% of the world population [1]. It is mainly driven by a dysregulated immune response and is primarily characterized by erythematous and scaly plaques on the skin [2]. This autoimmune disease is mediated by a complex pathogenesis involving environmental drivers, genetic triggers, and immune dysfunction [1,3]. The immune dysregulation is highlighted by excessive activation of the immune cells and expression of pro-inflammatory cytokines such as TNF-α, IL-17, or IL-23, which orchestrate the pathogenesis and severity of the disease [2,4,5].

Common therapeutic strategies include immunosuppressants and topical treatments such as methotrexate [6] or cyclosporine [7]. However, these compounds often combine limited long-term efficiency and numerous side effects [6,7,8,9]. Recent studies have highlighted the importance and efficacy of biologics for the treatment of psoriasis, which offer more limited side effects and more targeted approaches to control the dysregulated immune pathways [1,2,10]. Biologics targeting psoriasis are mainly fusion proteins or antibodies, such as TNF-α inhibitors (e.g., infliximab) or IL-17/IL-23 (e.g., secukinumab), that have important inhibitory effects in reducing psoriasis [2,3,6]. However, there is a constant need for new biologics and therapeutic approaches because some patients are refractory to specific treatments with biologics [2,3,11].

Natural compounds and bioactive molecules have enormous potential as modulators of inflammatory and immune cascades [12,13]. For instance, tick saliva presents a reservoir of bioactive compounds secreted to silence host immune defenses [14,15]. Among these bioactive compounds, serpins were reported for their pivotal and pleiotropic roles in modulation/suppressing host inflammatory and immune reactions through regulating the activity of key proteases [16,17]. Iripin-3, a salivary serpin from the European tick *Ixodes ricinus*, was reported as a pluripotent serpin with important immunomodulatory and anti-hemostatic activities that suppress the host anti-tick defenses [18].

In this study, an innate immunity-dependent mouse model with psoriatic inflammation induced by mannan [19,20] was used to explore the effects of Iripin-3 in psoriasis. This animal model mimics the different immune pathways involved in human psoriasis and allows the study of various compounds and their effects on disease development. By evaluating the role of Iripin-3 on the immune cascades involved in psoriasis, we aim to determine whether the tick salivary serpin can offer possible therapeutic advantages against psoriasis and other immune-mediated diseases.

## 2. Materials and Methods

### 2.1. Animals

Eight-to-twelve-week-old healthy BALB/c female inbred mice, purchased from Southern Medical University Experimental Animal Center, were maintained in a pathogen-free animal house and used in the experiments (approval number, CUMS-2018-0062-03). Mice were kept in an environment with 12 h light/dark cycles, and water and food were ad libitum. All animal procedures in this study were performed following the Laboratory Animals Welfare Act of Southern Medical University. The animal study was approved by the institutional review board of Southern Medical University (l2018183), Guangzhou, China, and performed according to the guidelines of the National Institutes of Health (NIH Publication No. 8023). The study was conducted in accordance with the local legislation and institutional requirements. Careful procedures were performed during the experiments to ensure the health of experimental mice by following the ARRIVE guidelines. Based on previous publications [20], we used 88 mice in total, and the detailed information is as follows: 40 mice for PASI scores, each group contained 10 mice and 4 groups. H&E/IHC/IF staining, flow cytometry/qPCR samples from the peak of psoriasis were obtained from the same batch of mice, with 6 mice per group and 4 groups. Terminal-period flow cytometry/qPCR samples were obtained from another batch of mice, with 6 mice per group across 4 groups. Three mice were excluded from the negative control because their total PASI scores on days 2–4 were significantly lower than those of the others. One mouse sample was excluded during the detection of CD11b^+^F4/80^+^ cell expression via flow cytometry because of the low cell numbers.

### 2.2. Cloning, Expression, and Purification of Iripin-3

Iripin-3 was expressed and purified, as previously reported [18]. Briefly, Iripin’s sequence was cloned into a pET-17b vector, and the resulting construct was transformed into *E. coli* BL21(DE3)pLysS cells. Expression was performed in LB medium in the presence of ampicillin (100 μg/mL) and chloramphenicol (34 μg/mL), and was started by adding 0.5 M IPTG. Iripin-3 was mainly present in inclusion bodies, which were isolated and dissolved in 6 M guanidine hydrochloride (pH 8) and 10 mM dithiothreitol. Following protein refolding, Iripin-3 was purified using gel filtration and ion exchange chromatography. Finally, endotoxins were removed using a detergent-based method by the company ARVYS Proteins, Inc. (Trumbull, CT, USA), and endotoxins were measured as reported in [21]. Endotoxin levels in the used Iripin-3 preparation were <0.01 EU/mg.

### 2.3. Mannan-Induced Psoriasis-like Inflammation Model and Samples Collection

In all the in vivo experiments, mice were randomly assigned to four groups using a random number table method: PBS (blank control), mannan (model group, Sigma-Aldrich), alpha-lactalbumin (negative control, Sigma-Aldrich), and Iripin-3 (treated group). Control and Iripin-3 treatment were performed without any selection bias. α-LA was used for negative control for Iripin-3 treatment, because it has a low molecular weight protein with stable properties and good solubility, and it does not cause inflammation. There are no positive controls for Iripin-3 treatment, which is a novel serine protease inhibitor, and no mature serine protease inhibitors are available. Each animal experimental group contained 6–10 mice. An estimated area of 2 cm x 3.5 cm was shaved at the back of mice after anesthesia with isoflurane (3%, RWD, Shenzheng, China). To induce psoriasis lesions, 5 mg mannan (100 μL/day, Sigma-Aldrich, St. Louis, MO, USA) and incomplete Freund’s adjuvant mixture (IFA, Sigma-Aldrich) were topically applied daily in a 1:1 ratio on a shaved area on the back of mice for three consecutive days as described earlier [22]. Control mice were treated with PBS. We performed the experiments with cystatins [20] and Iripin-3 at the same time with PBS and α-LA control groups. To monitor psoriasis development, the psoriasis area and severity (PASI) index was used [23]. Psoriasis lesions (redness, scales, and skin thickness) were scored on a scale from 0 to 4, where 0 indicated no symptoms, 1 mild, 2 moderate, 3 severe, and 4 very severe symptoms. Skin thickness was measured in the lesional areas using Vernier calipers (Neill-Lavielle, Louisville, KY, USA). The experiment is considered complete if any group of mice has a PASI score below 0.5 points. All mice treated with mannan developed skin inflammation. To examine the effect of Iripin-3 on skin inflammation, mannan-exposed mice were subcutaneously injected daily starting on day 0 to day 7 with 4 mg/kg Iripin-3, 4 mg/kg alpha-lactalbumin, or an equal volume of PBS. Based on previously published in vitro data [18], we used 4 mg/kg of Iripin-3, similar to other protease inhibitors from ticks published earlier by our group [20]. The double-blind method was used for drug administration, PASI scoring, and sample processing, with each step performed independently by different researchers.

Mice were sacrificed on day 4 or 7 to collect samples of skin, spleen, and inguinal lymph nodes. Skin samples were used for histology, skin immune cell and cytokine expression analysis, and draining lymph nodes, as well as spleen, were used for circulating immune cells characterization via flow cytometry. The back skin from each group was divided into four parts randomly for H&E, immunohistochemistry (IHC), immunofluorescence (IF) staining, and qPCR detection. Spleen and bilateral inguinal lymph nodes from each mouse were collected, and then ground into a cell suspension.

### 2.4. H&E and Immunohistochemistry Staining

The skin was fixed in 4% paraformaldehyde for 24 h at room temperature (RT). After overnight fixation, skin tissues were processed using standard fixing, dewaxing, and dehydration procedures. Then, the skin was washed in PBS and embedded in paraffin, with 8 μm sections cut using a paraffin slicer (Leica, Wetzlar, Germany). Sections were methanol-fixed, deparaffinized, dehydrated, and stained with hematoxylin solution (Phygene, Shanghai, China) or 0.2% eosin. Images were acquired at a 10× magnification using an Eclipse upright optical microscope digital camera (Nikon Ci-E, Tokyo, Japan). Epidermal thickness was evaluated using ten randomly selected regions per section. Baker’s score was used to judge pathological manifestations of psoriasis (Table 1) [22].

For IHC detection, paraffin skin sections were stained with biotin-conjugated anti-Ly6G antibodies and anti-F4/80 antibodies (1:100, Biolegend, CA, USA). Detection was performed with streptavidin-HRP (1:1000, Sigma-Aldrich) and DAB (3,3′-Diaminobenzidine) solution (Vector Laboratories, CA, USA). Sections were counterstained with hematoxylin before visualization with an optical microscope (Nikon).

### 2.5. Immunofluorescence Staining

Skin tissues were embedded in optimal cutting temperature (OCT) compound (Sakura Finetek Inc., CA, USA), snap-frozen, and stored overnight at −80 °C. Before embedding, samples were dehydrated with PBS containing 30% sucrose overnight and with 30% sucrose–PBS: OCT in a ratio of 1:1 gradually for 4 h. Tissues were sectioned (7 μm) using a cryostat and kept at −20 °C until used. The frozen sections were dehydrated in an ethanol series, fixed in acetone for 10 min, treated with 3% hydrogen peroxide, and blocked with 5% goat serum. Dendritic cells were stained with a biotin-anti-mouse CD11c antibody (1:100, Biolegend, California, USA) in combination with AF488-conjugated streptavidin (1:800, Biyotimes, Shanghai, CA). Nuclear DNA was detected by a DAPI (5 mg/mL, Biyotimes) staining for 5 min at RT. Confocal images were acquired using a Nikon Laser Confocal Microscope and then analyzed using an NIS Elements Viewer Imaging Software (Nikon).

### 2.6. Flow Cytometry

Cells were passed through a 70 μm cell strainer and washed with PBS three times. Cells isolated from the spleen were detected for macrophages (CD11b^+^F4/80^+^), dendritic cells (CD11b^+^CD11C^+^), and neutrophils (CD11b^+^ Ly6C/6G^+^) using the following fluorescent-labeled antibodies: PerCP-Cy 5.5 conjugated anti-F4/80 antibodies, PE-conjugated anti-CD11c antibodies, APC-conjugated anti-CD11b antibodies, and FITC-conjugated anti-Ly6c/6g antibodies (BD Biosciences, NJ, USA). The γδ T cells in draining lymph nodes were detected by staining with PerCP-Cy 5.5-conjugated anti-CD45 antibodies and PE-conjugated anti-γδ TCR antibodies (BD Biosciences) for 30 min at RT. To detect intracellular IL-17A expression, the obtained single-cell suspension from draining lymph nodes was lysed with red blood cell lysis buffer (Abcam, Cambridge, England) at RT for 5 min, and then cultured for 30 min with the PMA (Merck, Darmstadt, Germany) as well as ionomycin (Merck). Cells were first stained with APC-conjugated anti-mouse CD4 antibodies for 30 min at 4 °C in the dark, then fixed and permeabilized for 20 min at RT in the dark using a fixation/permeabilization solution (BD Biosciences). Later, cells were incubated for 30 min with PE-labeled anti-IL-17A antibodies. Samples were measured with an LSR II (BD Biosciences), and data were analyzed using the FlowJo Software Version 7.0 (Tree Star, CA, USA). For compensation, we used unstained samples to adjust the voltage of each fluorescence channel so that the peak of the negative group was 10^0^–10^1^ (logarithmic coordinates). To avoid interference from fluorescence, we used single-stained cells as controls. The gating strategies were shown in the flow cytometry figures.

To measure platelet–neutrophil and platelet–monocyte aggregates, whole human blood supplemented with 40 μM PPACK and 400 μg/mL Pefabloc FG was incubated with 3 μM Iripin-3 or an equal volume of TBS (pH = 7.4) as a control for 15 min at RT. Subsequently, samples were stained with an APC-conjugated anti-GPIbα antibody in combination with a PE-labeled anti-CD66c antibody or an AF488-labeled anti-CD14 antibody for 10 min, and then activated for five minutes with 30 μM TRAP-6 at 37 °C while shaking. Samples were fixed using BD Cytofix, and red blood cells were lysed with red blood cell lysis buffer containing 155 mM NH_4_Cl, 10 mM NaHCO_3_, and 0.1 mM EDTA (Abcam, Cambridge, England). Samples were measured with an Accuri C6 flow cytometer (BD Biosciences, Franklin Lakes, NJ, USA), and data were analyzed using the FlowJo software v.10.8.1 (BD Life Sciences, Ashland, OR, USA).

### 2.7. RNA Isolation and qRT-PCR

Total RNA was extracted from the skin lesions using the Trizol (Invitrogen, CA, USA), and the mRNA was isolated and dissolved in RNAse-free water (Promega, Madison, WI, USA). According to the manufacturer’s instructions, the mRNA samples were reverse-transcribed using the PrimeScript RT reagent Kit with gDNA Eraser (Thermo Scientific, Cambridge, UK). Quantitative RT-PCR reactions were performed using SYBR Premix Ex Taq II Tli RNaseH Plus (Takara biotech, Osaka, Japan) in a LightCycler 96 thermocycler (Roche, Basel, Switzerland). β-actin (Generay, Shanghai, China) was used as the housekeeping gene. For the amplification curve, the amplification efficiency was 97.8%. The amplicon sizes for all the primers were 18–24 bp. Specific base composition of primers is listed in Table 2. The Ct for β-actin is 16–18 cycles, for other primers it is 23–26 cycles. For the melting curve, the Tm was around 58–62 °C. The amplification program consisted of 1 cycle of 95 °C for 3 min followed by 45 cycles of 95 °C for 5 s, 55 °C for 5 s, and 72 °C for 10 s, and at the end, one cycle at 72 °C for 5 min. In order to show the differences between each group, PBS was used as a control for pro-inflammatory cytokines (TNF-α, IL-6, IL-22, IL-23, IL-17A/E/F). And Mannan was used for the control group for anti-inflammatory cytokines (IL-4, IL-10). And the relative fold inductions were then calculated using the 2^–ΔΔCt^ algorithm.

### 2.8. Statistical Analysis

**Figure 1:**
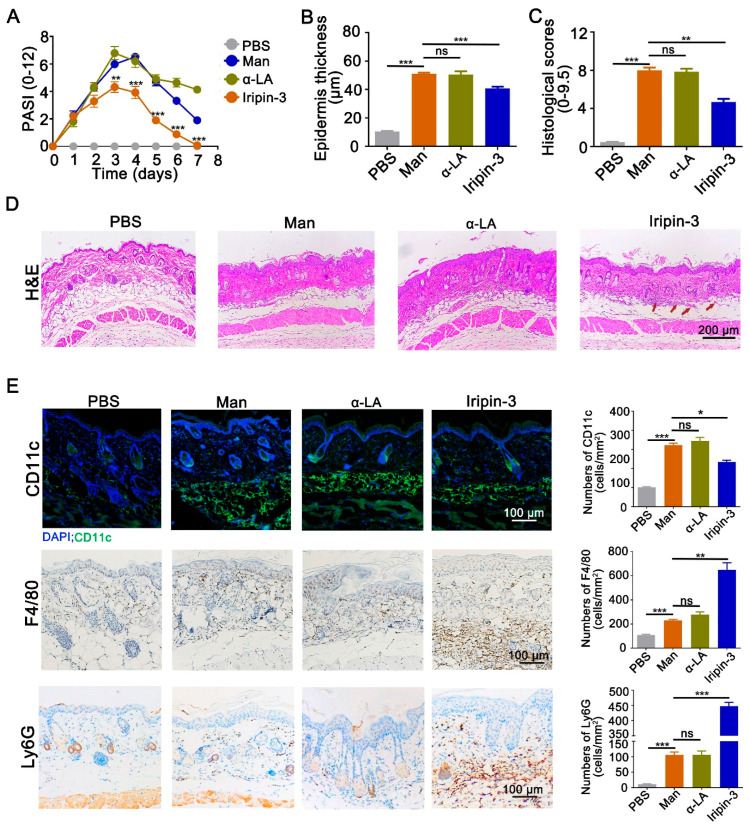
**Iripin-3 decreases mannan-induced psoriasis-like inflammation in mice.** Using a mouse model of mannan-induced psoriasis-like inflammation, the immunomodulatory properties of Iripin-3 were studied. All mice developed psoriasis-like inflammation. The severity of the psoriasis lesions was evaluated using the PASI scores during the experiment (n = 10 mice per group) (**A**). Skin samples were harvested at the peak of psoriasis (day 4) and stained with H&E. Epidermis thickness was measured (**B**), and the Baker’s score was calculated to judge the pathological manifestations of the psoriasis-like lesions (**C**) (n = 6 mice per group). (**D**) Shown are representative images of the H&E staining (scale bar = 200 µm). A higher number of blood vessels was observed in the Iripin-3-treated skin (Figure 3, marked by red arrows). (**E**) The infiltration of immune cells positive for CD11c, F4/80, and/or Ly6G in the skin lesions was assessed using immunofluorescence (CD11c) and immunohistochemistry (F4/80, Ly6G). Representative pictures (scale bar = 100 µm) and corresponding quantifications of the number of cells positive for CD11c, F4/80, or Ly6G per mm2 (n = 6 mice per group) are shown. Data are depicted as mean ± SEM. * *p* < 0.05; ** *p* < 0.01; *** *p* < 0.001 compared to the mannan group (unpaired *t*-test). Man: mannan, α-LA: negative control alpha-lactalbumin.

**Figure 2:**
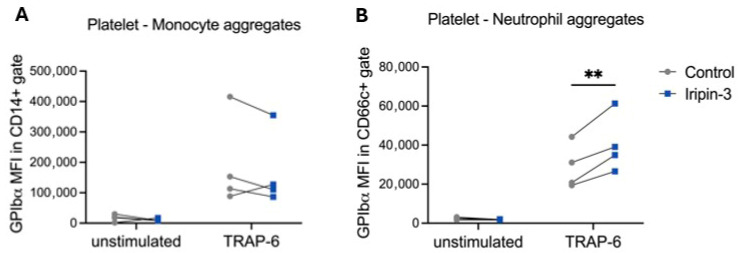
**Iripin-3 increases platelet–neutrophil aggregation.** Citrate-anticoagulated whole blood was incubated with 3 μM Iripin-3 or equal volumes of vehicle for 15 min at RT. After samples were stained with antibodies directed against GPIbα and CD66c or CD14, platelet–neutrophil and platelet–monocyte aggregation were induced by 30 μM TRAP-6 at 37 °C and assessed upon fixation using flow cytometry. Shown is the mean fluorescence intensity (MFI) of the GPIbα staining of cells positive for CD66c (**A**) or CD14 (**B**). Data are depicted as mean ± SD, n = 4. * *p* < 0.05; ** *p* < 0.01; *** *p* < 0.001. (Repeated measures two-way ANOVA with Šídák’s multiple comparisons test).

**Figure 3:**
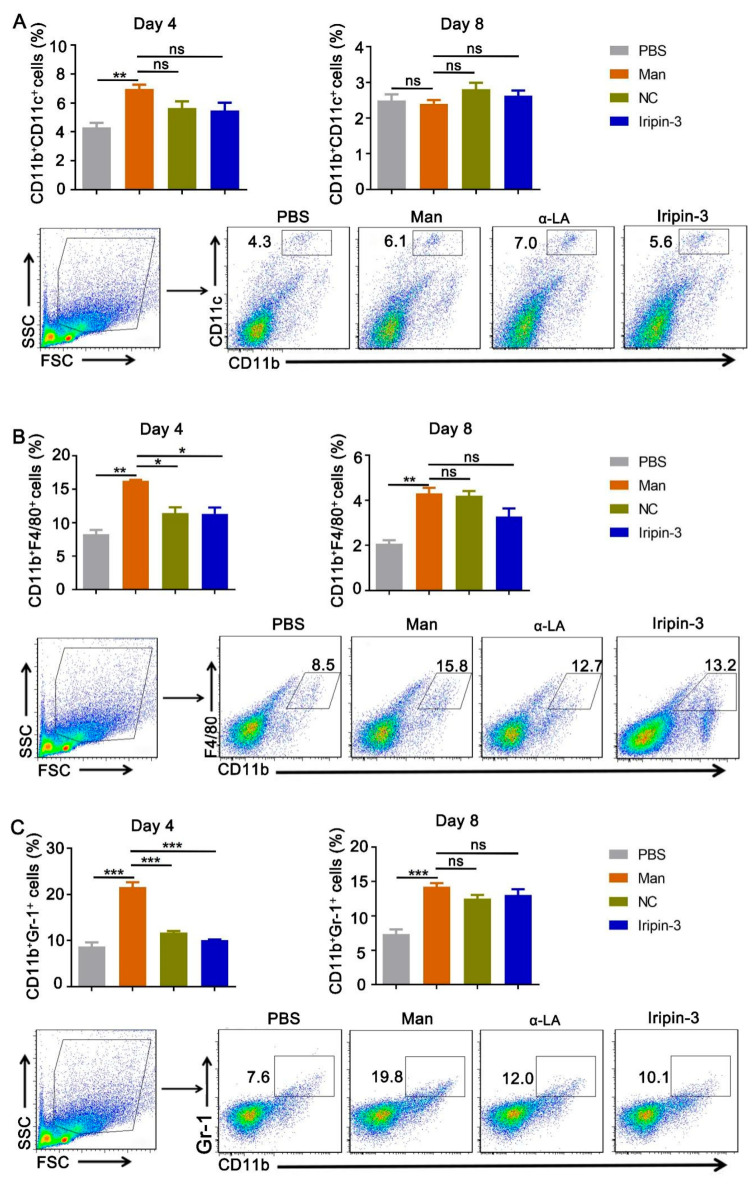
**Iripin-3 treatment decreased the number of macrophages and neutrophils in the spleen.** On days 4 and 8, the spleen samples were collected, and immune cells were isolated. Using flow cytometry, the percentage of (**A**) dendritic cells (CD11b^+^, CD11c^+^), (**B**) macrophages (CD11b^+^, F4/80^+^), and (**C**) neutrophils (CD11b^+^, GR-1^+^) was quantified, and representative dot plots on day 4 are shown. Data are depicted as mean ± SEM, n = 6 mice per group. * *p* < 0.05; ** *p* < 0.01; *** *p* < 0.001 compared to the mannan group (unpaired *t*-test). Man: mannan, α-LA: alpha-lactalbumin.

**Figure 4:**
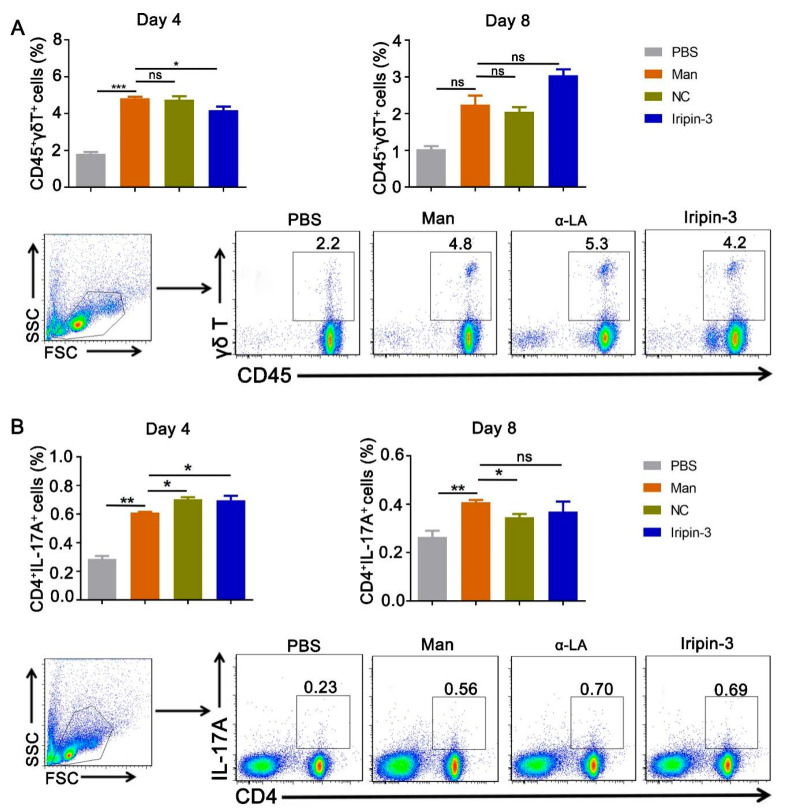
**Iripin-3 alters the number of CD45^+^γδ T and CD4^+^IL-17A^+^ cells in the lymph nodes.** Draining lymph nodes were harvested on days 4 and 8, and immune cells were isolated. The percentage of (**A**) γδ T (CD45^+^γδ TCR^+^) and (**B**) Th17 (CD4^+^IL-17A^+^) cells was determined using flow cytometry, and representative dot plots on day 4 are shown. Data are depicted as mean ± SEM, n = 6 mice per group. * *p* < 0.05; ** *p* < 0.01; *** *p* < 0.001 compared to the mannan group (unpaired *t*-test). Man: mannan, α-LA: alpha-lactalbumin.

**Figure 5:**
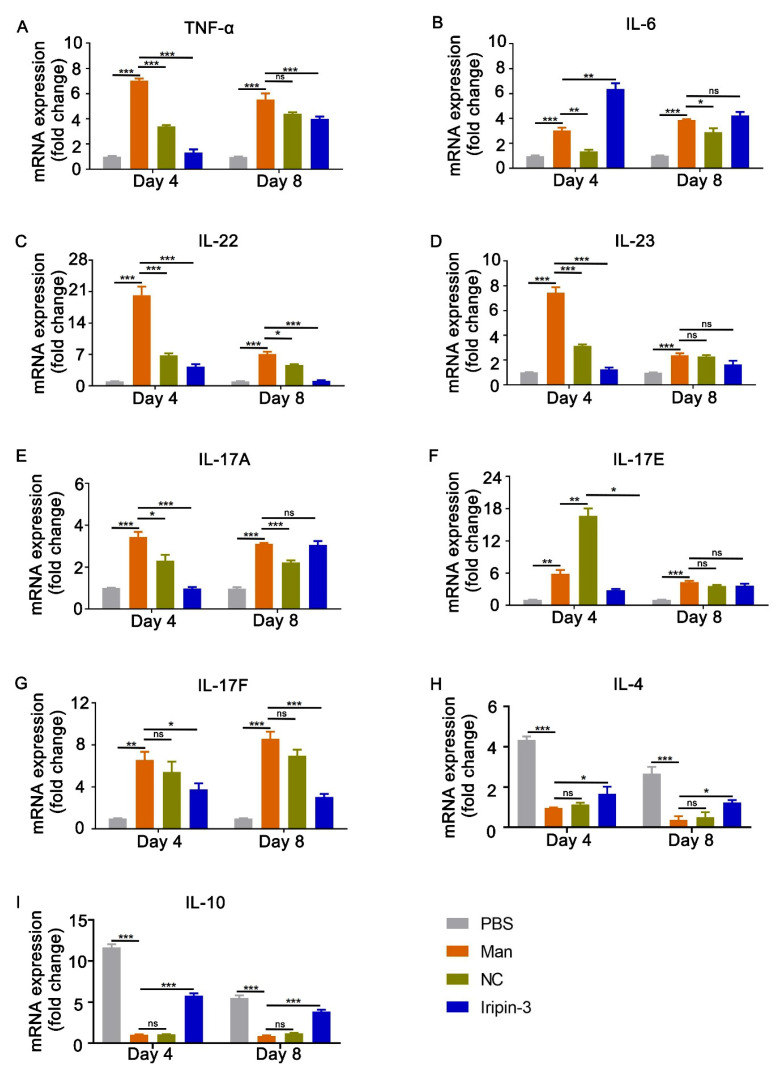
**Iripin-3 reduces mannan-induced skin inflammation by affecting the IL-23/IL-17A axis.** Skin samples were collected on days 4 and 8, and the mRNA samples were extracted using TRIzol. The expression of TNF-α (**A**), IL-6 (**B**), IL-22 (**C**), IL-23 (**D**), IL-17A (**E**), IL-17E (**F**), IL-17F (**G**), IL-4 (**H**), and IL-10 (**I**) was evaluated using q-PCR. Data are depicted as mean ± SEM, n = 6 mice per group, and qPCR analyses were repeated twice. (**A**–**G**) The PBS group was normalized to 1, and the (**H**,**I**) mannan group was normalized to 1 to show the difference between each group. * *p* < 0.05; ** *p* < 0.01; *** *p* < 0.001 compared to the mannan group (unpaired *t*-test). Man: mannan, α-LA: alpha-lactalbumin. Table 3 resumes the mean fold-changes for each cytokine, timepoint, and treatment.

Data was analyzed using GraphPad Prism 8 software, and results in Figures 1 and 3–5 are presented as mean ± SEM, and Figure 2 is shown as mean ± SD. Two-tailed unpaired Student’s *t*-test was used for comparisons between 2 groups, and one-way analysis of variance with Bonferroni or Newman–Keuls correction was used for multiple comparisons. Probability values ≤ 0.05 were considered significant. *, *p* < 0.05; **, *p* < 0.01; ***, *p* < 0.001 and NS, not significant. Error bars depict SEM/SD with a 95% confidence interval.

## 3. Results

Iripin-3 was earlier shown to modulate adaptive immune responses [18]. Based on the pleiotropic character of tick serpins [17,22,24,25] and to further investigate the effect of Iripin-3 on innate immune responses, the tick salivary serpin was studied in a mouse model of psoriasis-like inflammation dependent on innate immune responses. Psoriasis-like inflammation was induced by the topical application of mannan, a robust model of psoriasis induction [19]. Iripin-3 was subcutaneously injected daily over the timespan of the experiment, while the administration of α-lactalbumin was used as a negative control.

### 3.1. Iripin-3 Decreases Mannan-Induced Psoriasis-like Inflammation

To evaluate the severity of mannan-induced skin inflammation (MISI) over the time course of the experiment, the psoriasis area and severity index (PASI) was calculated daily and used to monitor disease symptoms, including redness, scales, and thickness, with a maximum score of 12. As evident from the increase in PASI over the first three days, all mice treated with mannan developed skin inflammation (Figure 1A). Administration of Iripin-3 resulted in a significant decrease in PASI from days 3 to 7, and the mice returned to near normal, similar to control on day 7 (PASI = 0 ± SEM) (Figure 1A). At the peak of MISI (day 4), Iripin-3 administration led to a significant decrease in epidermal thickness and Baker’s score (Figure 1B,C), as shown in representative pictures of H&E staining (Figure 1D), suggesting a dampening effect of Iripin-3 on skin inflammation.

A closer evaluation of the innate immune response in the skin of mannan-treated mice revealed a reduction in the number of dendritic cells, whereas the number of F4/80^+^ macrophages and Ly6G^+^ neutrophils infiltrated in the inflamed skin was increased upon subcutaneous Iripin-3 injection at the peak of MISI (day 4) (Figure 1E). Several reports highlighted the close association between psoriasis pathogenesis and platelet activation [26,27,28,29,30]. Thus, we tested the in vitro effect of Iripin-3 on platelets, neutrophils, and platelet–monocytes in an attempt to explain the observed increase in neutrophils in the lesional site. Figure 2A,B show that platelet–monocyte aggregates were not affected in the presence of Iripin-3 (Figure 2A), while the presence of the serpin significantly increased the neutrophil–platelet aggregates (Figure 2B). The latter finding was performed for human samples, and thus, no conclusions can be drawn until testing samples from mice.

### 3.2. Dendritic Cells and γδ T Cells in Secondary Lymphoid Organs Contributed to Decreased Psoriasis After Iripin-3 Treatment

To show the effect of Iripin-3 on inflammatory cells in secondary immune organs, we detected innate immune cells (dendritic cells, macrophages, and neutrophils) in the spleen using multi-color flow cytometry. We identified that the relative number of dendritic cells (CD11b^+^CD11c^+^ cells) was decreased by Iripin-3 treatment (Figure 3A) while it downregulated the percentages of CD11b^+^F4/80^+^ macrophages and CD11b^+^Gr-1^+^ neutrophils (Figure 3B,C) expression in the spleen on day 4 in MISI. However, Iripin-3 has no effects on dendritic cells, macrophages, and neutrophils at the end of psoriasis (day 8). Considering the importance of γδ T and Th17 cells in the development of psoriasis [4,31], we assessed the number of inflammatory γδ T and Th17 cells in the draining lymph nodes on days 4 and 8. In comparison to the mannan group, Iripin-3 treatment decreased the expression of γδ T cells (CD45^+^γδ TCR^+^) at the peak of MISI but rather caused an increase in the relative number of Th17 cells (CD4^+^ IL-17A^+^) (Figure 4A,B). On the other hand, Iripin-3 could not affect the number of γδ T and Th17 cells at the end of psoriasis. Gating strategies and representative pictures of innate immune cells from each group are shown in Figure 3 and Figure 4.

### 3.3. Iripin-3 Decreased Mannan-Induced Skin Inflammation by Affecting the IL-23/IL-17 Axis

Next, we determined how Iripin-3 affected the cytokine response in the skin lesions. To this end, mRNA was extracted from skin tissues at the peak and the end of MISI (days 4 and 8), and the mRNA expression of the pro-inflammatory, psoriasis-related cytokines TNF-α, IL-6, IL-22, IL-23, IL-17A, IL-17E, and IL-17F was measured. Anti-inflammatory cytokine expression was also measured by assessing the expression dynamics of IL-4 and IL-10. Compared to the untreated mice, mice receiving Iripin-3 injections showed a reduced expression of TNF-α on days 4 and 8 (Figure 5A). Remarkably, the expression of IL-6 was strongly increased at the peak of MISI but not at the end of psoriasis after Iripin-3 treatment (Figure 5B). Regarding IL-22, Iripin-3 significantly downregulated their gene expression at the peak and end of MISI (Figure 5C). This observation is important as IL-22 decrease is highly correlated to keratinocyte proliferation [32], while IL-23 mRNA decrease indicates that dendritic cells are involved in driving the disease progression [33]. And Iripin-3 downregulated the mRNA expression of IL-23 at the peak of psoriasis (Figure 5D). A significant decrease was also observed for the expression of the Th17 family of cytokines on day 4 (IL-17A, IL-17E, and IL-17F), while Iripin-3 had no effects on IL-17A and IL-17E mRNA expression on day 8 (Figure 5E,F). And anti-inflammatory cytokines IL-4 and IL-10 were significantly increased after Iripin-3 treatment (Figure 5H,I) in this study using a mannan-induced psoriasis mouse model.

**Table 3:** Summary of mean mRNA fold-changes for TNF-α, IL-6, IL-17A/E/F, IL-22, IL-23, IL-4, and IL-10 from mice treated with PBS, Man, NC, or Iripin-3 on days 4 and 8.

Cytokine	Day 4 PBS	Day 4 Man	Day 4 NC	Day 4 Iripin-3	Day 8 PBS	Day 8 Man	Day 8 NC	Day 8 Iripin-3
TNF-α	1.0	7.0	3.4	1.3	1.0	5.5	4.3	4.0
IL-6	1.0	3.0	1.4	6.3	1.0	3.9	2.9	4.2
IL-22	1.0	20.3	6.8	4.3	1.0	7.1	4.6	1.1
IL-23	1.0	7.4	3.2	1.3	1.0	2.4	2.3	1.6
IL-17A	1.0	3.6	2.3	1.0	0.9	3.2	2.3	3.1
IL-17E	1.0	5.8	16.7	2.8	1.0	4.3	3.6	3.6
IL-17F	1.0	6.5	5.4	3.8	1.0	8.6	7.0	3.0
IL-4	4.4	1.0	1.1	1.7	2.6	0.4	0.6	1.3
IL-10	11.3	1.0	1.0	5.8	5.4	0.9	1.2	3.8

## 4. Discussion

Our results show that Iripin-3 treatment decreased PASI scores (redness, scales, and thickness), both at the peak and at the end of psoriasis, contributing to the reduction in epidermal thickness and histological scores, which correlated with the modulated keratinocyte functions, infiltration of immune cells, and modulation of cytokine levels.

In psoriasis, keratinocytes, as the primary effector cells, are abnormally activated and secrete inflammatory cytokines to recruit immune cells [34]. Upon receiving signals from inflammatory cells, these cells undergo abnormal hyperproliferation and impaired differentiation, leading to acanthosis and the formation of scaly plaques [35]. IL-17, IL-22, and TNF-α act synergistically in psoriatic lesions, with IL-17 directly driving excessive keratinocyte proliferation and recruiting immune cells [36], while IL-22 is the primary factor inducing epidermal hyperplasia and suppressing normal keratinocyte differentiation [32]. TNF-α not only amplifies inflammation via the IL-23/IL-17 axis, creating a positive feedback loop, but also induces angiogenesis [37]. Interestingly, Iripin-3 treatment significantly inhibited the expression of these cytokines, which could explain the decrease in inflammation, thickness, and scales after Iripin-3 treatment. To explore the effects of Iripin-3 on keratinocytes, the levels of IL-6 in lesional skin were detected. However, our results show that Iripin-3 did not decrease IL-6, although Iripin-3 was shown earlier to decrease its expression in bone marrow-derived macrophages [18]. We have detected a higher number of blood vessels in the Iripin-3-treated skin by histology, and the increased IL-6 production might be from the endothelial cells of blood vessels. It should be noted that IL-6 is a pleiotropic cytokine, and it is closely linked to the healing of skin wounds as well [38,39], by plausibly modulating the stratum corneum regeneration and skin barrier functions [40]. It should also be noted that IL-6 has both pro- and anti-inflammatory properties. IL-6 can not only facilitate an acute inflammation but also control it by suppressing the level of pro-inflammatory cytokines like TNF-α and IL-1 [41,42] and increasing anti-inflammatory factors like interleukin 1 receptor antagonist [43] and interleukin 10 [44], depending on the cellular context, with different signaling pathways, classical versus trans signals determining its effect. Therefore, an increase in IL-6 might also indicate an attempt to reestablish homeostasis in the epidermal permeability barrier functions because of the disease-ameliorating functions of Iripin-3.

Additionally, we analyzed the expression of immune cells in the lesional skin and secondary lymphoid organs after Iripin-3 treatment. Our results show that Iripin-3 could decrease the expression of dendritic cells in the lesional skin, macrophages, and neutrophils in the spleen at the peak of psoriasis. However, an increase in macrophages and neutrophils in the lesional skin was observed after Iripin-3 treatment. A possible explanation for the increased number of neutrophils is the ability of platelets to assist neutrophils in the tissue infiltration [30], and the increased neutrophil–platelet aggregates in the presence of Iripin-3 could be through an effect on platelets, neutrophils, or both. However, the recombinant Iripin-3 used in our experiments was not glycosylated, which might have an impact on the anti-inflammatory and immunomodulatory activities of Iripin-3 mediated by its binding to cell surfaces and soluble immune mediators [18]. For example, only glycosylated, but not the non-glycosylated α-1-antitrypsin, was capable of binding IL-8, thus inhibiting IL-8-CXCR1 interactions [45], and IL-8 is important for neutrophil recruitment [46]. The increase in neutrophils may also be caused by an increase in the level of inflammatory factors such as IL-17 (Figure 5 E,G), which can promote the recruitment of neutrophils from blood vessels to the epidermis [36]. Additionally, recent research indicates that neutrophils are not a homogeneous population. Under specific conditions, other scientists observed the development of neutrophil subtypes with anti-inflammatory or pro-repair functions. These subtypes may promote inflammation resolution by releasing anti-inflammatory mediators and increasing the number of macrophages phagocytosing apoptotic cells. Recently, it was shown that siglec-F+ neutrophils promoted M2-type macrophage differentiation [47]. Therefore, it is possible that neutrophilia after Iripin-3 treatment may represent part of the inflammation resolution process, directly correlating with the observed decrease in disease severity.

An increase in F4/80+ cells in the lesional skin after Iripin-3 treatment might be due to an increase in M2 macrophages, which were shown to have a downregulatory role in psoriasis and psoriatic arthritis inflammation [48]. F4/80 is a widely used marker for identifying macrophages, particularly in mice, including both M1 and M2 subtypes. Other markers like CD11c, CD206, CD80, CD86, and CD163 are needed to further classify macrophage subtypes [49], but we did not use these specific markers; therefore, it is possible that the macrophage increase observed after Iripin-3 treatment might be due to an increase in M2-like macrophages. More experiments are needed to clarify these findings in the near future.

Iripin-3 treatment also increased the levels of IL-4 and IL-10. These cytokines are involved in anti-inflammatory functions and macrophage polarization from M1 to M2. Both these cytokines can also act on mononuclear cells and inhibit the production and secretion of pro-inflammatory cytokines like IL-23 and IL-17, leading to inflammation resolution [50,51]. IL-23 is primarily produced by myeloid immune cells such as dendritic cells, which plays a pivotal role in disease progression by driving the IL-17/IL-22 pathway and directly inducing epidermal hyperplasia in psoriasis [52,53]. The inhibition of this cytokine correlated with a decrease in dendritic cells after Iripin-3 treatment, which indicates that Iripin-3 could downregulate the recruitment and cytokine secretion of dendritic cells in psoriasis. In addition, we have also investigated the effects of Iripin-3 on γδT and Th17 cells, which are crucial for IL-17 family cytokines secretion in psoriasis, and exert the crosstalk between keratinocytes and other innate immune cells [33].

Our data show that Iripin-3 decreased the expression of γδT cells but increased the percentage of Th17 cells at the peak of psoriasis. Meanwhile, Iripin-3 also decreased the mRNA expression of IL-17 family cytokines (IL-17A, IL-17E, and IL-17F) on day 4. Beyond the traditionally recognized Th17 cells, innate immune cells like γδ T cells emerge as a primary and significant producer of IL-17. Activation of TL1A receptors present on γδ T cells in the dermis by IL-23 produces IL-17 and IL-22 [54], which in turn directly drives abnormal proliferation of keratinocytes and inflammatory responses [31]. The above observations indicate that Iripin-3 could reduce the expression of IL-17 family cytokines by suppressing the number of γδ T cells. It should also be noted that not all Th17 cells are pro-inflammatory [55], and some of the Th17 cells can have regulatory roles under specific conditions in psoriasis [56]. The mechanism for serine protease inhibitor Iripin-3 to improve psoriasis is multi-targeted and multi-level, not simply and uniformly suppressing all IL-17-producing cells. The reasons are the ability of Iripin-3 to inhibit kallikrein, matriptase, trypsin, plasmin, thrombin, and FVIIa. It also reduced survival of mouse splenocytes, impaired proliferation of CD4+ T cells, suppression of the Th1 immune responses and induction of Treg [18]. All of these mechanisms could potentially be contributing to the decrease in localized tissue damage and inflammatory cascades observed in the dermis, which may directly influence γδ T cells secreting IL-17. On the other hand, other Serpins (SerpinB7, SerpinA12) were shown to inhibit overactive serine proteases and hyperproliferation of keratinocytes, as well as promote the repair of the skin, but without directly affecting Th17 cells [57].

Earlier, we showed that Iripin-3, a serine protease inhibitor from salivary glands of the tick *Ixodes ricinus*, could reduce adaptive immune responses by inhibiting the activity of matriptases and kallikreins [18]. Matriptases, highly expressed in psoriatic lesions, can indirectly activate inflammatory pathways within keratinocytes, leading to the production of inflammatory cytokines, including IL-1β, IL-6, and TNF-α [58]. Kallikrein-catalyzed release of bradykinin and other kinins directly regulates the functions of keratinocytes by acting on bradykinin B1 and B2 receptors [59]. The above observations indicate that Iripin-3 may inhibit keratinocyte proliferation and cytokine secretion by suppressing these enzymes, which in turn could lead to a decrease in the number of dendritic cells and IL-23, thereby diminishing γδ T cell recruitment, secretion of pro-inflammatory cytokines, and inflammation. Understanding the precise mechanisms and the structure–function studies of the anti-inflammatory effects of Iripin-3 requires further pre-clinical studies and clinical trials. If proven, Iripin-3 could represent a novel bio-inspired therapeutic for psoriasis treatment targeting the IL-23/γδ T/IL-17 axis.

## Figures and Tables

**Table 1:** Baker’s scores of H&E staining.

Feature	Scores
Munro’s small abscess in stratum corneum	2
Hyperkeratosis	0.5
Hypokeratosis	1
Thinning/disappearing of granular layer in epidermis	1
Thickening of spinous layer	1
Skin protuberances and undulations	0.5–1.5 *
Infiltration of mononuclear or multinuclear cells	0.5–1.5 *
Upper mastoid	0.5
Telangiectasia	0.5

* Based on the severity.

**Table 2:** Primer pairs for quantitative RT-PCR analysis.

Target Gene	Forward Primer	Reverse Primer	Accession Number
β-actin	ACCGTGAAAAGATGACCCAG	GTACGACCAGAGGCATACAG	NM_007393
TNF-α	ACGCTCTTCTGTCTACTGAACT	ATCTGAGTGTGAGGGTCTGG	NM_013693
IL-6	GAGAAAAGAGTTGTGCAATGGC	CCAGTTTGGTAGCATCCATCAT	NM_001314054
IL-17A	CCCCTAAGAAACCCCCACG	TAAAGTCCACAGAAAAACAAACACG	NM_010552
IL-17E	ACAGGGACTTGAATCGGGTC	TGGTAAAGTGGGACGGAGTTG	NM_080729
IL-17F	GTCAGGAAGACAGCACCA	AGCCAACTTTTAGGAGCA	NM_145856
IL-22	CATGCAGGAGGTGGTACCTT	CAGACGCAAGCATTTCTCAG	XM_006513865
IL-23-P19	AGCAACTTCACACCTCCCTAC	ACTGCTGACTAGAACTCAGGC	NM_031252
IL-4	GTCATCCTGCTCTTCTTTCTCG	TTGGCACATCCATCTCCGT	NM_021283
IL-10	GGCCTTCCCTACTTCACAAG	GGCCTTCCCTACTTCACAAG	NM_000572

## Data Availability

The authors confirm that the data supporting the findings of this study are available within the article. The raw data that support the findings of this study are available from the corresponding authors, upon reasonable request.
